# Prevalence and Aetiology of Left Ventricular Thrombus in Patients Undergoing Transthoracic Echocardiography at the University of Maiduguri Teaching Hospital

**DOI:** 10.1155/2014/731936

**Published:** 2014-09-29

**Authors:** Mohammed Abdullahi Talle, Faruk Buba, Charles Oladele Anjorin

**Affiliations:** ^1^Cardiology Unit, Department of Medicine, University of Maiduguri Teaching Hospital, PMB 1414, Maiduguri 600001, Nigeria; ^2^Department of Medicine, College of Medical Sciences, University of Maiduguri, PMB 1069, Maiduguri 600001, Nigeria

## Abstract

*Objectives*. We sought to determine the prevalence and aetiology of LVT among patients undergoing echocardiography. *Methods*. We reviewed case notes and echocardiographic data of patient diagnosed with LVT using noncontrast transthoracic echocardiography. Definition of various conditions was made using standard guidelines. Mean ± SD were derived for continuous variables and comparison was made using Student's *t*-test. *Results*. Total of 1302 transthoracic echocardiograms were performed out of which 949 adult echocardiograms were considered eligible. Mean age of all subjects with abnormal echocardiograms was 44.73 (16.73) years. Abnormalities associated with LVT were observed in 782/949 (82.40%) subjects among whom 84/782 (8.85%) had LVT. The highest prevalence of 39.29% (33/84) was observed in patients with dilated cardiomyopathy, followed by myocardial infarction with a prevalence of 29.76% (25/84). Peripartum cardiomyopathy accounted for 18/84 (21.43%) cases with some having multiple thrombi, whereas hypertensive heart disease was responsible for 6/84 (7.14%) cases. The lowest prevalence of 2.38% (2/84) was observed in those with rheumatic heart disease. Left ventricular EF of <35% was recorded in 55/84 (65.48%). *Conclusions*. Left ventricular thrombus is common among patients undergoing echo, with dilated cardiomyopathy being the most common underlying aetiology followed by myocardial infarction. Multiple LVTs were documented in peripartum cardiomyopathy.

## 1. Introduction

The development of left ventricular thrombus (LVT) is a well-known complication in various cardiac conditions with the highest rate observed in acute anterior myocardial infarction and congestive heart failure (CHF) as a result of severe left ventricular (LV) systolic dysfunction [1, 2].

The prevalence of LVT, especially in settings where early percutaneous coronary intervention (PCI) facilities are available, is seemingly reducing with estimates ranging between 5% and 15% [2, 3]. Solheim et al., reported an incidence of 15% within 3 months of acute myocardial infarction (AMI) in selected patients managed with primary PCI [4]. However, Rabbani et al. found that the incidence of LVT remains persistently high (35%) in spite of PCI for AMI involving the anterior wall [5]. On the other hand, the occurrence of LVT in cardiomyopathy and CHF ranges from 10 to 30% [6, 7].

The constellation, of endothelial injury, hypercoagulability, and blood stagnation, which are well described previously as Virchow's triad, is responsible for the formation of thrombus [8, 9]. In AMI, other predisposing factors are large infarct size, severe apical asynergy, LV aneurysm and anterior myocardial infarction (MI) [10]. The early and active recognition of LVT is vital to avert the unwanted sequelae of systemic thromboembolic events [1, 8, 9].

Transthoracic echocardiography (TTE) is still the widely employed modality in diagnosis of LVT because of its access, safety, and convenience. It has been demonstrated to have sensitivity of 90–95% and specificity of 85–90% in a setting of adequate imaging [11, 12] when compared to findings at surgery and autopsy. However, care must be exercised to exclude false positive results occasioned by endocardial elastosis, trabeculae, false tendons near-field clutter, and artefacts among others [13, 14]. The detection rate can be enhanced by off-axis imaging planes in situations where substrates for the development of LVT such as abnormal wall motion and dilated ventricles with markedly reduced ejection fraction (EF) exist.

Improved LV cavity assessment and thrombi detection using TTE contrast studies were noted to be better than noncontrast TTE, especially for mural (laminar) and small thrombus [15]. This led to the recommendation that echo contrast should be employed when noncontrast images are suboptimal for clear-cut diagnosis [16]. Additional advantages of echocardiography include the determination of LV chamber size, demonstration of regional wall motion abnormalities, and detection of Doppler-derived transmitral flow changes. Lower mitral* E* wave deceleration time and abnormal wall motion score index were both associated with LVT formation [17].

In this retrospective study, we reviewed demographic data and aetiology of LVT in patients undergoing echocardiography at the University of Maiduguri Teaching Hospital over a three-year period.

## 2. Methodology

We reviewed case notes and echocardiographic data of patient diagnosed with LVT using noncontrast transthoracic echocardiography at the University of Maiduguri Teaching Hospital, Maiduguri (UMTH), Nigeria, from January 2011 through December 2013. Data form was designed comprising patients' age, gender, associated comorbidities, and echocardiographic indices.

The echocardiographic procedures were performed with MyLab 50CV (Esaote) and Siemens Acuson X300 with variable 1.7–2.2 MHz transducer to ensure adequate imaging analyses. The diagnosis of LVT was reached when a mass was noted adjacent to the myocardium on multiple plane views throughout the cardiac cycle and associated regional wall motion abnormalities [1, 13]. Regional wall motion abnormality was considered present if hypokinesis, akinesis, and dyskinesis were observed in at least two segments of the left ventricular wall or if left ventricular aneurysm is observed, while old infarct was diagnosed in the presence of segmental thinning, LV remodelling and fibrosis [13]. Left ventricular dyssynchrony was diagnosed in the presence of a delay >130 ms between maximum posterior excursion of the septum and peak anterior excursion of the posterior LV wall on M-mode [18]. Off-axis imaging planes were used where the standard imaging planes did not show LVT despite high index of suspicion. Left ventricular dimensions were determined by the leading-edge to leading-edge method and determination of left ventricular ejection fraction (EF) was based on the recommendations of American Society of Echocardiography (ASE) and European Society of Echocardiography [19]. Left ventricular diastolic filling patterns were recorded using the mitral inflow pulsed-wave Doppler examination. Average values of* E*- and* A*-filling velocities, their ratio (*E*/*A*), and the deceleration time of* E*-wave velocity were determined from 3 to 5 consecutive cardiac cycles.

Dilated cardiomyopathy was diagnosed in the presence of globular LV with LVIDD of >56 mm, LV sphericity index ratio of less than 1.5 and EF < 40% [20]. Diagnosis of myocardial infarction was based on the combination of documented history of chest pain, ECG abnormalities, and segmental wall abnormalities [21]. Peripartum cardiomyopathy was diagnosed on the basis of temporal relation of heart failure to last pregnancy and delivery as proposed in ESC guideline [22]. Hypertensive heart disease (HHD) was diagnosed in hypertensive patients in the presence of concentric/eccentric left ventricular hypertrophy or concentric left ventricular remodeling, left atrial dilatation and/or systolic, and/or diastolic left ventricular dysfunction [23]. Diagnosis of rheumatic heart disease (RHD) was made using the World Heart Federation criteria [24].

The data was analysed using SPSS v 16.0 Chicago, IL, USA. Mean ± SD was derived for continuous variables and comparison was made using Student's* t*-test. A* P* value of <0.05 was considered significant. The conduct of this study was approved by the Research and Ethics Committee of the UMTH as part of the Heart Failure Registry.

## 3. Results

A total of 1302 transthoracic echocardiograms were performed from January 2011 through December 2013. Seventy eight (5.99%) studies were excluded due to incomplete imaging, inconclusive data, and poor image quality. Of the remaining 1224 echocardiograms, 162/1224 (13.24%) were reported as normal studies. One hundred and thirteen (10.64%) of the abnormal images were paediatric studies, while 949 (89.36%) were adult studies. All subjects included in this study came from various parts of the northeast geopolitical zone of Nigeria, where UMTH serves as the major referral tertiary hospital.

The 949 abnormal adult echocardiograms reviewed were made of 463/949 (48.79%) males and 486/949 (51.21%) females. The mean age of all subjects with abnormal echocardiograms was 44.73 (16.73) years compared to 45.43 (13.44) for subjects with LVT. (*P* = 0.71). Male subjects with LVT were significantly older than the females (50.90 ± 9.84 versus 40.21 ± 14.42, *P* = 0.001). Abnormalities associated with LVT were observed in 782/949 (82.40%) of subjects. The demographic and echocardiographic profile of subjects with and without LVT is illustrated in Table 1.

**Figure 1:**
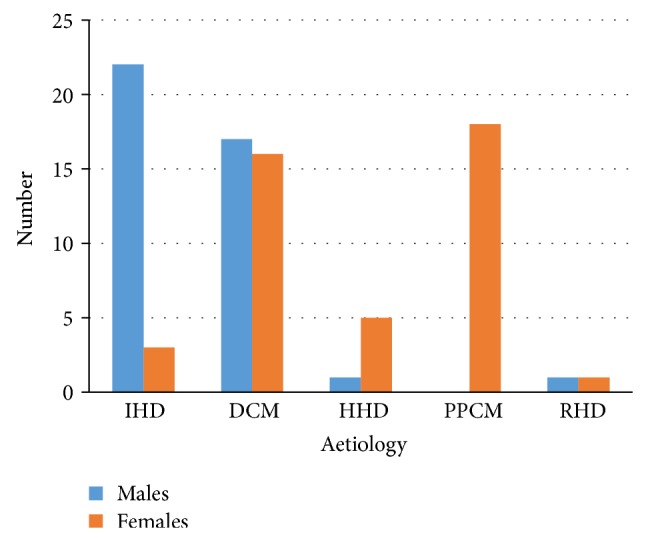
Distribution of aetiology of left ventricular thrombus by gender. IHD = ischaemic heart disease; DCM = dilated cardiomyopathy; HHD = hypertensive heart disease; PPCM = peripartum cardiomyopathy; RHD = rheumatic heart disease.

Eighty four (8.85%) cases of LVT were identified. Forty one (4.32%) was recorded in males while 43/84 (4.53%) cases were recorded in females. Off-axis (free-style) imaging was used in identifying 12/84 (14.29%) cases. The highest prevalence of 39.29% (33/84) was observed in patients with dilated cardiomyopathy, followed by MI with a prevalence of 29.76% (25/84). Eighty seven percent of the cases of MI involved the anterior wall, with the remaining 13% involving the inferior and the inferior-posterior region. Of the patients having LVT following MI, old infarction was documented in 11/25 (44%), whilst 10/25 (40%) presented within the first week of the onset of symptoms. Only 4/25 (16%) of patients with MI presented with LVT within 24 hours of onset of symptoms. Eighty eight percent (22/25) of cases of myocardial infarction were observed in males. Peripartum cardiomyopathy accounted for 18/84 (21.43%) of the LVT, whereas HHD was responsible for 6/84 (7.14%) cases. The lowest prevalence of 2.38% (2/84) was observed in those with RHD. A gender-based distribution of the different aetiologies of LVT is illustrated in Figure 1.

**Figure 2:**
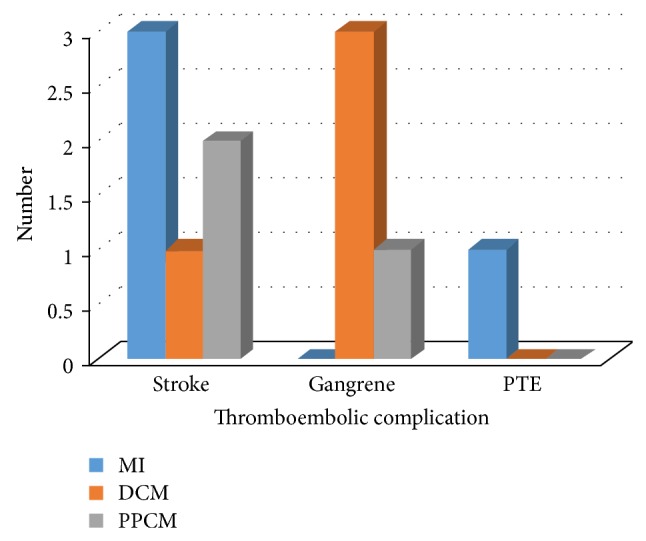
Presenting thromboembolic complications among patients with left ventricular thrombus. DCM = dilated cardiomyopathy; MI = myocardial infarction; PPCM = peripartum cardiomyopathy; PTE = pulmonary thromboembolism.

Among the 84 subjects with LVT, 11 (13.1%) presented for echocardiography with thromboembolic complications (Figure 2). There were 6/11 (54.5%) cases of stroke in patients with MI (50.0%), PPCM (33.3%), and DCM (16.7%). Peripheral gangrene was present in 4/11 (36.4%) patients with DCM (75.0%) and PPCM (25.0%). One patient (9.1%) with MI and biventricular thrombus presented with pulmonary thromboembolism without features of deep venous thrombosis.

Nine (81.8%) of the subjects with thromboembolic complications presented with solitary apical thrombus while 2/11 (18.2%) had multiple LVT of varying sizes. All subjects presenting with thromboembolic complications from PPCM and DCM had an EF < 35%.

Treatment modalities were identified in 76/84 (90.5%) including all cases that presented with thromboembolic phenomenon. These patients were treated using anticoagulant doses of low molecular weight heparin (enoxaparin), unfractionated heparin (for patients unable to afford enoxaparin), and warfarin adjusted to achieve therapeutic INR.

Four (36.4%) of the patients that presented with thromboembolic complications (a case of stroke and two cases of gangrene and a case of pulmonary embolism) died during hospitalization. One subject with peripheral gangrene had an above the knee amputation, whilst one left the hospital against medical advice and died at home.

Data on follow-up echocardiography at one month was identified in only 36/84 (42.9%) with 16/36 (44.4%) cases of complete resolution of LVT. Level of adherence and duration of anticoagulation could not be conclusively ascertained.

The mean left ventricular end diastolic dimension in subjects with LVT was 60.45 mm (7.65) with 69/84% (82.14%) having dilated left ventricular end diastolic dimension >56 mm. The mean left ventricular EF was 28.83% (9.64). Left ventricular EF of <35% was recorded in 55/84 (65.48%) while 40/84 (47.6%) had an EF < 30%. Seventy eight (92.86%) of the subjects with LVT had an EF of <50%. Regional wall motion abnormality was recorded in 29/84 (34.52%), while left ventricular dyssynchrony was documented in 16/84 (19.05%). Left ventricular hypertrophy was documented in 12/84 (14.29%). Left ventricular apical aneurysm was observed in 1/84 (1.19%) subject while pseudoaneurysm with apical LVT was identified in 1/84 (1.19%). Echocardiographic profile of the subjects is illustrated in Table 2.

**Figure 3:**
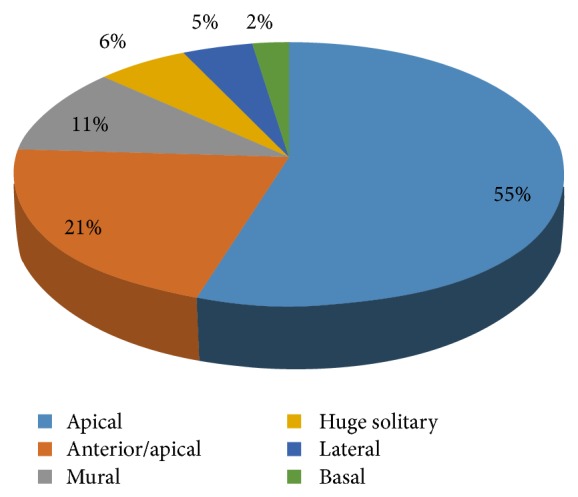
Locations of left ventricular thrombus observed.

**Figure 4:**
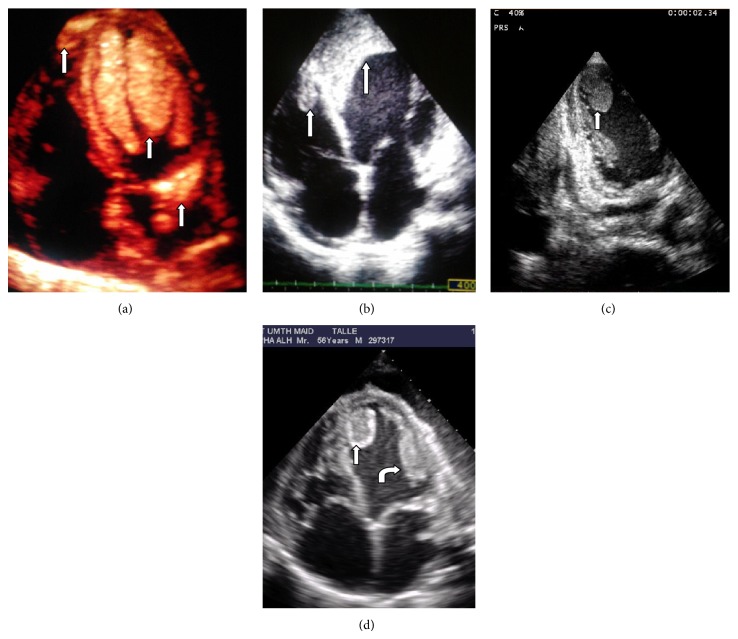
Images showing thrombus (white arrows) in various locations. (a) Biventricular and left atrial thrombi in a patient with PPCM. (b) Biventricular thrombi following anteroapical MI. (c) Off-axis plane showing apical LVT. (d) Multiple LVT with central lucency and LV spontaneous echo contrast in DCM.

The various locations of LVT are illustrated in Figure 3. Multiple apical LVTs were recorded in 24/46 (52.17%) cases, whilst 11/46 (23.91%) of those with apical LVT had coexisting right ventricular thrombus. The highest rate of biventricular thrombus was observed among patients with DCM (45.5%) and PPCM (27.3%) with EF < 30%. Coexisting left and right atrial thrombi were each identified in 2/46 (4.35%). Huge solitary LVTs were identified in subjects with DCM and PPCM, whereas extensive anterior/apical LVTs were observed among subjects with myocardial infarction and extensive regional wall motion abnormalities. The distribution of the various locations of LVT and representative images obtained from the subjects are illustrated in Figures 3 and 4.

## 4. Discussion

We report a prevalence of 8.85% for left ventricular thrombus among patients with abnormal echocardiogram over a three-year period at the University of Maiduguri Teaching Hospital. This to our knowledge is the first report on the prevalence of LVT among patients undergoing echocardiography from the northern part of Nigeria. Seventy six percent of LVT in our series involved the apical segments, in keeping with reports from other studies. The apex is the most common region involved in LVT in patients with MI as well as nonischemic cardiomyopathy.

The highest prevalence of 39.3% was recorded among patients with nonischaemic DCM. This is in keeping with previous reports by other workers [25, 26]. Dilated cardiomyopathy is associated with dilatation of both left and right ventricles with reduced systolic function. The resultant biventricular stasis promotes the formation of thrombus, most frequently in the left ventricle, followed by the right ventricle. The risk of peripheral embolization is reported to be high in the setting of LVT [8, 9]. Coexisting right ventricular thrombus (resulting in biventricular thrombus) was documented in 15.2% of subjects with DCM and LVT. Biventricular involvement was observed in those with severe systolic dysfunction (EF < 30%). Previous studies reported increased LVIDD and low ejection fraction to be independent predictors of LVT formation [26, 27]. This is demonstrated in our subjects with the overwhelming majority having increased LVIDD and low EF.

Myocardial infarction accounted for the second highest prevalence of 29.8%. Left ventricular thrombus is a well-documented complication of myocardial infarction, with prevalence varying from 60% in the prethrombotic era to between 5% and 15% in settings where early PCI is instituted [2, 3, 28]. However, Sani et al. reported a lower prevalence of 6.7% for echo-detected LVT among patients with myocardial infarction at Kano in north western Nigeria [29]. Determinants of LVT formation following MI include the region and extent of involvement, formation of left ventricular aneurysm, and extent of systolic dysfunction among others [4, 5, 10]. Most of the subjects with LVT in our study had anterior/apical involvement with dilated LV and reduced EF. This finding concurs with reports of higher prevalence of LVT following AMI compared to nonanterior MI from other centres [2]. Although there are reports indicating reduced incidence of LVT with thrombolytic therapy and PCI, the majority of patients in our series presented with unrecognized MI resulting in heart failure. The few presenting with AMI came outside the time frame for the administration of thrombolytics and PCI was not available. These might have contributed to the increased prevalence of LVT.

More cases of myocardial infarction were recorded in male subjects, reflecting the higher prevalence of coronary artery disease among males compared to females. Involvement of the anterolateral wall results in large area of wall motion abnormality, favouring stasis and thrombus formation [8]. A combination of wall motion abnormalities, LV dilatation with reduced EF, and inherent risks of thrombosis associated with the development of MI contributes to the formation of LVT. Although aneurysms are potent contributors to formation of LVT following MI, only one case each of aneurysm and pseudoaneurysm was documented.

Peripartum cardiomyopathy in our series had LVT prevalence of 21.4%. This is similar to what was reported by Sliwa et al. among black patients at Baragwanath Hospital [30], but higher than the 12.3% reported in Sokoto, north western Nigeria [31]. Karaye and Sani reported a higher prevalence of 54.6% among patients with PPCM in Kano [32]. The variation in rates reported by the different studies may be due to differences in severity of LV dilatation and dysfunction in the population studied. Concomitant right ventricular thrombus (resulting in biventricular thrombus) was observed in 16.7% of our subjects with PPCM and LVT. Biventricular thrombus in the setting of PPCM is largely limited to case reports, buttressing the rarity of such occurrences [33–35]. The prevalence of 16.7% for biventricular thrombus in PPCM in this study is rather high. This may be attributed to the advanced state of ventricular dysfunction, as well as late presentation. The formation of thrombus in the setting of PPCM is attributed to stasis consequent upon the poor myocardial contractility as well as the hypercoagulable state of pregnancy which may persist for up to six weeks postpartum [36].

Although HHD constituted the most common diagnosis among the cases reviewed, the prevalence of LVT was 7.1%, all occurring in those with impaired LV systolic function. Information on LVT complicating HHD without MI or profound global hypokinesis is scarce. The role of hypertension in enhancing prothrombotic or hypercoagulable state by impacting on all components of the Virchow's triad, termed the thrombotic paradox of hypertension or the Birmingham paradox, was reported by Lip [37]. In the study of the Framingham Offspring Study, Poli and colleagues reported an association between blood pressure and plasma PAI-1 and tPA antigen levels, suggesting impaired fibrinolysis with increasing blood pressure [38]. In another study, Preston et al. demonstrated elevated markers of endothelial and platelet activation that could result in procoagulant effect [39].

Rheumatic heart disease is an uncommon cause of LVT in our series. The prevalence of 2.3% was observed in two patients having severe chronic rheumatic mitral regurgitation with dilated and poorly contractile ventricles. We speculate that the LVT observed in these patients is a result of the increased LVIDD and low EF rather than the rheumatic aetiology of valve lesion. Intracardiac thrombus in the setting of RHD is commonly observed in left atrium and left atrial appendage of patients with mitral stenosis [40], better visualized using TEE.

About 13% of all subjects with LVT had thromboembolic complications at the time of presentation for echocardiography, mainly in patients with myocardial infarction and DCM. Stroke was the commonest presenting thromboembolic phenomenon among subjects with myocardial infarction, whilst peripheral gangrene predominated in those with DCM. Embolic complications were reported in about 10% of cases of MI in the prethrombolytic era [41]. The overwhelming majority of our patients with MI presented late, precluding the administration of thrombolytic agents. Although reports on factors associated increased risks of embolism are inconsistent [8], characteristics associated with heightened propensity for embolization include protrusion into LV cavity, large thrombus size, diffuse LV dilatation, and impaired systolic function, among others [26]. Our subjects presenting with thromboembolic complications exhibited many of these features. Despite having the majority of our subjects placed on anticoagulant therapy, poor follow-up data made it impossible to assess outcome of treatments.

Despite the advancements in the area of LVT assessment with the use of contrast agents, TTE is widely accepted as the primary screening tool for LVT in clinical practice, especially when the procedure is tailored to detection of LVT with use of multiple imaging planes including off-axis (free-style) imaging [42]. Off-axis imaging plane was used in identifying 14.3% of LVT in our study. In addition, TTE has the added advantage of higher sensitivity/specificity for apical LVT compared to TEE.

The catastrophic thromboembolic risks of LVT may be reduced by appropriate treatment of MI using thrombolytics and, where available, PCI. Guidelines on antithrombotic therapy and prevention of thrombosis recommend the use of warfarin in patients with anterior MI and LVT, or high risk for LVT, as well as patients with systolic dysfunction and documented LVT [43]. Given the high prevalence of LVT in patients with DCM, MI, and PPCM, we recommend a focused assessment for LVT using TTE in subjects presenting with any of them and severe systolic dysfunction of other aetiologies. This is particularly important in poor resource settings where contrast echo is not available. Patients with LVT should be treated with antithrombotic agents in line with existing guidelines to prevent thromboembolic complications [43].

Our study has a number of limitations. Being a retrospective study, we are unable to assess the outcome of LVT in the subjects. Given the higher accuracy of contrast echo for the detection of LVT, the prevalence reported in our study may be an underestimated, as some cases of laminar and small thrombi might have been missed out. Similarly, echocardiographic studies might not have been focused on detection of LVT in some cases, resulting in reduced yield. Our diagnosis of IHD is not supported by determination of cardiac markers. Assessment of other risk factors associated with intracardiac thrombosis has not been done, making assessment of causality rather inconclusive. However, the aim of our study was to determine the prevalence of LVT among patients undergoing transthoracic echo in our centre.

## Figures and Tables

**Table 1:** Baseline characteristics and comparison of disorders in subjects with and without left ventricular thrombus.

Characteristics	With LVT, *n* = 84	Without LVT, *n* = 698	*P* value
Age, yr	45.43 ± 16.7	44.73 ± 16.73	0.71
Female	43 (51.2)	370 (53.0)	0.95
Male	41 (48.8)	328 (47.0)	0.96
DCM	33 (39.3)	180 (25.8)	0.17
MI	25 (29.8)	56 (8.0)	0.03
PPCM	18 (21.4)	53 (7.6)	0.24
HHD	6 (7.1)	301 (43.1)	0.18
RHD	2 (2.4)	108 (15.5)	0.99

LVT = left ventricular thrombus; DCM = dilated cardiomyopathy; MI = myocardial infarction; PPCM = peripartum cardiomyopathy; HHD = hypertensive heart disease; RHD = rheumatic heart disease.

**Table 2:** Echocardiographic profiles of subjects with and without left ventricular thrombus.

Parameter	With LVT, *n* = 84	Without LVT, *n* = 698	*P* value
Mean LVDD (mm)	60.45 (7.65)	55.57 (10.91)	0.001
LVDD > 56 mm	69 (82.14%)	437/698 (62.61%)	0.001
Mean EF	28.83 (9.64)	46.35 (17.11)	0.001
EF < 30	40 (47.62%)	119/698 (17.05%)	0.001
EF < 35	55 (65.48%)	223/698 (31.95%)	0.001
RWMD	29 (34.52%)	56/698 (8.02%)	0.001
LV dyssynchrony	16 (19.05%)	45/698 (6.45%)	0.001

LVIDD = left ventricular internal diameter in diastole; EF = ejection fraction; RWMD = regional wall motion defect; LV = left ventricle; LVT = left ventricular thrombus.
